# A continuously changing selective context on microbial communities associated with fish, from egg to fork

**DOI:** 10.1111/eva.13027

**Published:** 2020-06-09

**Authors:** Nicolas Derome, Marie Filteau

**Affiliations:** ^1^ Institut de Biologie Intégrative et des Systèmes (IBIS) Université Laval Québec QC Canada; ^2^ Département de Biologie Université Laval Québec QC Canada; ^3^ Département des Sciences des aliments Institut sur la nutrition et les aliments fonctionnels (INAF) Université Laval Québec QC Canada

**Keywords:** antimicrobial alternatives, aquaculture, biotic and abiotic factors, evolutionary forces, food microbiology, microbial interactions, microbiota

## Abstract

Fast increase of fish aquaculture production to meet consumer demands is accompanied by important ecological concerns such as disease outbreaks. Meanwhile, food waste is an important concern with fish products since they are highly perishable. Recent aquaculture and fish product microbiology, and more recently, microbiota research, paved the way to a highly integrated approach to understand complex relationships between host fish, product and their associated microbial communities at health/disease and preservation/spoilage frontiers. Microbial manipulation strategies are increasingly validated as promising tools either to replace or to complement traditional veterinary and preservation methods. In this review, we consider evolutionary forces driving fish microbiota assembly, in particular the changes in the selective context along the production chain. We summarize the current knowledge concerning factors governing assembly and dynamics of fish hosts and food microbial communities. Then, we discuss the current microbial community manipulation strategies from an evolutionary standpoint to provide a perspective on the potential for risks, conflict and opportunities. Finally, we conclude that to harness evolutionary forces in the development of sustainable microbiota manipulation applications in the fish industry, an integrated knowledge of the controlling abiotic and especially biotic factors is required.

## 1 INTRODUCTION

In response to the human population growth, demand for fish proteins has dramatically increased and will undoubtedly continue to rise along this century, especially as fish nutritional value is becoming highly coveted (FAO, 2018). In response to the increase of fish demand, the industry is turning mainly to aquaculture production (OECD & FAO, 2015) which is accompanied by serious challenges. On the one hand, aquaculture presents severe ecological risks. For instance, most aquaculture systems operate on coastlines or near inland rivers or ponds, thus exerting a major impact on natural habitat biodiversity and productivity. In addition, the high density of farmed stock increases exposure to unprecedented disease outbreaks (Llewellyn, Boutin, Hoseinifar, & Derome, 2014), causing significant losses in aquaculture (FAO, 2016). Meanwhile, current methods of sanitary control, mostly relying on chemicals including antibiotics, are currently reaching their limits both in terms of efficiency and sustainability (Cabello et al., 2013; Derome, 2019). The growing concern of antibiotic resistance has led multiple countries to ban or restrict the use of antibiotics for livestock production (Vieco‐Saiz et al., 2019). A wide diversity of genes families conferring resistance against 24 antibiotic types were detected in the gut microbiome of farmed fish (Tyagi, Singh, Billekallu Thammegowda, & Singh, 2019), underlining that the use of antibiotics should be avoided in aquaculture. Indeed, aquaculture systems are deemed genetic hotspots for gene transfer owing to their high diversity of bacteria, their combined exposure history to antimicrobial compounds and often their proximity to other farming activities (Watts, Schreier, Lanska, & Hale, 2017). Thus, to become truly sustainable, the aquaculture industry has no choice but to adopt alternative strategies to control disease occurrence and promote optimal host–microbiota functional interactions (Derome, 2019). On the other hand, fish is among the most perishable food products and can rapidly become a health hazard through microbial growth and chemical change (FAO, 2016). Fish spoilage mechanisms include chemical oxidation, enzymatic reactions, but most importantly in fresh products, microbial activity (Boziaris & Parlapani, 2017). Given that 27% of fish worldwide are being lost or wasted along the food distribution chain (FAO, 2016), and that up to 25% of losses in the fishery industry can be attributed to microbial growth and activity (Wiernasz et al., 2020), improvement of product preservation strategies is strongly needed. However, these strategies will have to meet the consumer demands for products that are minimally processed and free from artificial additives (Asioli et al., 2017). Thus, for both of these microbial‐based challenges affecting the fish industry, natural and sustainable strategies to promote fish health and growth in aquaculture as well as quality and safety of fish products are required.

Given the fundamental role microorganisms play both in the production and transformation of fish commodities, their microbial community ecology has captured the attention of scientists. From the animal host perspective, colonization by microorganisms is a dynamic process, called microbiota ontogeny, which is increasingly interpreted under a community ecology framework as an ecological succession in fish (Abdul Razak & Scribner, 2020; Burns et al., 2016; Sylvain & Derome, 2017). Through the applications of community ecology principles to microbiota studies, microbiota ontogeny in fish was evidenced to be influenced by both neutral and non‐neutral evolutionary forces (Cheaib, Seghouani, Ijaz, & Derome, 2020; Heys et al., 2020; Sloan, Woodcock, Lunn, Head, & Curtis, 2007; Stegen, Lin, Konopka, & Fredrickson, 2012). In its food counterpart, microbial contamination of the flesh is also akin to an ecological succession, but the theoretical aspects remain elusive in this context, although some foods have been suggested as tractable models to study microbial ecosystems (Wolfe & Dutton, 2015).

At the same time, on both sides of the fish industry, applications involving microbiota manipulations are being developed such as the use of prebiotics (Guerreiro, Oliva‐Teles, & Enes, 2017) and probiotics (Banerjee & Ray, 2017; Chauhan & Singh, 2019; Choudhury & Kamilya, 2018; Hoseinifar, Sun, Wang, & Zhou, 2018; Jahangiri & Esteban, 2018), bioprotective cultures (Ben Said, Gaudreau, Dallaire, Tessier, & Fliss, 2019), bacteriocin applications (Sahoo, Jena, Patel, & Seshadri, 2016; Vieco‐Saiz et al., 2019; Wang, Zhang, Ouyang, & Li, 2019) and others. However, their ecological repercussions along the food chain are underrecognized. For instance, microorganisms present on the host and its environment contribute to the initial product contamination (Gram & Huss, 1996); thus, microbiota manipulation in aquaculture may have consequences on fish product shelf‐life and safety. Moreover, the existence of an interplay between microbial community structure and rapid evolution has been proposed, which could have important repercussions for microbiological applications (O’Brien, Hodgson, & Buckling, 2013). Indeed, applications such as probiotic treatment have been shown to alter microbial community structure (Gupta, Fečkaninová, et al., 2019), but the microevolution that can occur on ecological timescales has not been investigated so far.

In light of these recent advances, how can we harness evolutionary forces in the development of sustainable microbiota manipulation applications in the fish industry? In this work, we try to answer this question from the interdisciplinary point of view of an evolutionary biologist and a food scientist, both trained in the Bernatchez Lab (Box 1). We first discuss the particular context of the fish microbiota of living hosts and food commodities and the evolutionary forces shaping microbial community assembly, summarizing the selectively changing context along the production chain. Then, we discuss the strategies to modulate microbial communities in aquaculture and the food industry from an evolutionary standpoint and highlight of the potential for risks, conflict and opportunities.

### BOX 1 Personal reflections on our time in the Bernatchez Lab

During an undergrad internship in the thriving scientific environment of the Bernatchez Lab, I was introduced to high‐throughput experiments and discovered a passion for data analysis, which defined my whole scientific background. Years later, as a postdoctoral researcher, Louis knew just the right challenge to assign me to keep me motivated. The most valuable scientific insight I gained then is to reflect on how things are connected to each other in a systems network perspective. This is ultimately how I became interested in biological interactions, which now translates into the microbial interaction thematic of my own laboratory—Marie Filteau, laboratory member 2003 and 2012.

The most important thing I have learned in the Bernatchez Lab, as a postdoctoral researcher, is to be always confident with the outcome of a project, as well as being creative and, perhaps most importantly, intuitive. Also, I fully realized the benefits of collaborative research, and the importance of team social events for which Louis paid a particular attention. What I have appreciated the most was the unconditional support I received, which allowed me to push my limits further. This valuable experience I gained in the Bernatchez Lab still inspires me as a PI—Nicolas Derome, laboratory member 2003‐2006.

## 2 FISH MICROBIOTA FROM LIVING HOST TO PRODUCT

Microorganisms are an important part of the fish life and shelf‐life, constituting first the host microbiota and then the product contamination. The host–microbiota system refers to the host organism and the microbial communities, encompassing bacteria, archaea, eukaryotes (protozoa, fungi, …) and viruses, which colonize its different body surfaces (e.g. skin, gills, intestine). The members of these transdomain microbial communities tightly interact with each other and with their host, thus exerting a pervasive role in regulating the host physiology homeostasis (Llewellyn et al., 2014; McFall‐Ngai et al., 2013). For instance, the zebrafish model (*Danio rerio*, Cyprinidae) revealed tight functional interactions between the host and its microbiota such as metabolism regulation, immune system development and maturation, and *via* the vagus nerve, brain development and various behaviours (Davis, Bryda, Gillespie, & Ericsson, 2016; Rawls, Samuel, & Gordon, 2004). This host–microbiota interaction is highly dynamic and particularly influential at early life stages as pioneering microbes will fine‐tune epigenetic patterns of the host organism (reviewed in Gerhauser, 2018), therefore exerting long‐term effects on its physiological phenotype and, in turn, on its overall fitness (Bäckhed, Ley, Sonnenburg, Peterson, & Gordon, 2005).

The pioneering microbiota colonizes body surfaces (egg chorion, skin mucus, digestive tract mucosa and gills) by two main processes: bacteria and other microorganisms are either transmitted vertically (Sylvain & Derome, 2017) or horizontally, from the environment (reviewed in Llewellyn et al., 2014). The latter is especially true for aquatic oviparous species, eggs being surrounded by aquatic microbial communities before hatching and the subsequent opening of their digestive tract, whereas strict viviparous species are first exposed to the maternal environment before their first contact with the environmental water. Interestingly, matrotrophic viviparity, which consists of *intra‐utero* maternal feeding via uterine mucosal secretion, was reported in surfperches (Longo & Bernardi, 2015) and rockfish (Boehlert & Yoklavich, 1984). However, aside from rockfish (Kim, Hwang, & Kwon, 2001), viviparous fish species are mostly grown for the ornamental aquarium industry (e.g. Poeciliidae), such that vertical transmission of microbial symbionts might play a very limited role in microbial succession in farmed fish. This is especially true as postfertilization egg disinfection is a common practice in aquaculture to prevent opportunistic disease (reviewed in Assefa & Abunna, 2018), which may potentially disrupt transmission of maternal symbionts (Lauzon et al., 2010). Overall, the host colonization by microorganisms is a dynamic process, called microbiota ontogeny. Along microbiota ontogeny, biotic factors, such as host genotype (Boutin, Sauvage, Bernatchez, Audet, & Derome, 2014; Dionne, Miller, Dodson, Caron, & Bernatchez, 2007; Rawls, Mahowald, Ley, & Gordon, 2006; Uren Webster, Consuegra, Hitchings, & Garcia de Leaniz, 2018; Zoetendal, Akkermans, Akkermans‐van Vliet, de Visser, & de Vos, 2001), life stage cycle (Llewellyn et al., 2016; Lokesh, Kiron, Sipkema, Fernandes, & Moum, 2019; Nagpal et al., 2017; Sylvain & Derome, 2017) and population density (Dehler, Secombes, & Martin, 2017; Landeira‐Dabarca, Sieiro, & Álvarez, 2013; Llewellyn et al., 2014; Sylvain et al., 2016), as well as abiotic factors such as water chemistry, temperature, nutrition and xenobiotics including antibiotics (Oliveira et al., 2016; Sylvain, Holland, Audet‐Gilbert, Luis Val, & Derome, 2019), are shaping the host microbiota, either in an additive or synergistic way. Given that the pioneering microbiota exerts a significant impact on the host physiology (e.g. genetic expression regulation) during the early life stages (i.e. larvae, juveniles), it is expected that exposure to contrasting abiotic factors will induce distinct physiological phenotypes (Goodrich, Davenport, Clark, & Ley, 2017). Furthermore, these effects can persist even if the causative factor is removed. In a mouse model exposed to antibiotics, for instance, although microbiota taxonomic composition was observed to recover after exposure, the metabolic phenotypes persisted, thus stressing the importance of microbiota ontogeny early steps in growth and development (Cox et al., 2014). The further understanding of the complex relationships between fish hosts and their associated and environmental microbial communities is strongly needed to model the disruption of the host–microbiota immunological equilibrium, which delineates the frontier between health and disease.

The host microbiota will ultimately influence the food product characteristics by contributing to its initial contamination (Gram & Huss, 1996). Microorganisms found on fresh fish come from the host microbiota, its environment at the time of capture and strains present in the processing environment (Boziaris & Parlapani, 2017), as well as from human manipulations (Leroi, 2010). The transition from host to product microbiome can be seen as an ecological succession, that is a change in species composition following a disturbance, in particular the primary succession of a newly created habitat (Tipton, Darcy, & Hynson, 2019). Indeed, in healthy hosts, the flesh is considered to be sterile (Leroi, 2010; Svanevik, Levsen, & Lunestad, 2013), but the muscle can become contaminated by microorganisms during capture, storage and transformation. Therefore, this newly created niche may harbour both co‐evolving (stable) interactions and naïve relationships, where the partners have not met before.

The initial microbiota of fish product spans a taxonomically wide range of bacteria, but the change in extrinsic parameters may select for few microorganisms able to proliferate and outcompete the others (Boziaris & Parlapani, 2017). Even in fresh, minimally processed fish, food preservation conditions are different from the natural aquatic environment of the fish, potentially altering its interactions with other members of the microbiota. Importantly, animal and food products represent contrasting ecological niches in their composition; gut microbes are often specialists, and cross‐feeding is a strong force shaping its composition (Gutiérrez & Garrido, 2019) while in foods common nutrients are available, relaxing selective pressures related to energy sources utilization. Fish is rich in nonprotein nitrogen compounds such as creatine, nucleotides, trimethylamine oxide, free amino acids and dipeptide. Combined with its high pH and water activity, these characteristics make fish particularly prone to microbial deterioration by fast‐growing bacteria (Leroi, 2010). This contrast between animal and food environment may explain why endogenous gut contaminants are often displaced by exogenous contaminants during spoilage (Chaillou et al., 2015). Ultimately, fish origin, processing and storage conditions, as well as microbial interactions, combine to define the resulting spoilage microbiota of fish (Boziaris & Parlapani, 2017). Another factor to consider about spoilage is that causative microorganisms (i.e. specific spoilage organisms, SSO) are not always dominant in the microbial community of an altered product. For instance, it was reported that although *Pseudomonas* is often dominant in sensorially altered products, it is not a good indicator of quality (Silbande et al., 2018). Besides, spoilage may come from specific microbial interactions within the community. For instance, lactic acid bacteria, *Brochothrix thermosphacta* and diverse Gram‐negative bacteria can form consortia causing fish deterioration (Cifuentes Bachmann & Leroy, 2015; Silbande et al., 2018). Moreover, the outcome of different species combination on spoilage can vary from enhancement to inhibition (Silbande et al., 2018). SSO presence depends on various factors including the fish species and its origin, and the transformation and conservation parameters of the product (Boziaris & Parlapani, 2017). To complicate matters, food spoiling behaviours have been shown to be strain‐dependent (Cocolin & Ercolini, 2015; Leroi, 2010). For these reasons, many studies have focused on specific bacteria rather than on whole community analysis. Further study of SSO isolates’ behaviour in a community context may reveal the biotic and abiotic dependencies of spoiling activity, which could be related to common life‐history traits.

During their often short shelf‐life, fresh fish products can be perceived as microbial ecosystems that are not subject to the same evolutionary scale as the living host (McMeekin et al., 2008). Nevertheless, food contamination and the subsequent spoilage is a heterogeneous process involving complex microbial communities that are only beginning to be characterized at the community level with the use of new generation molecular tools (Chaillou et al., 2015; Cocolin & Ercolini, 2015; De Filippis, Parente, Zotta, & Ercolini, 2018; Parente, Zotta, Faust, De Filippis, & Ercolini, 2018).

## 3 EVOLUTIONARY FORCES DRIVING FISH MICROBIOTA ASSEMBLIES

Stochastic (i.e. neutral) and deterministic (i.e. non‐neutral) processes underpin fish‐associated microbiota structure and assembly. In community ecology, taxonomic drift and dispersal are stochastic processes referring to evolutionary forces like genetic drift and migration, respectively. Deterministic processes refer to competitive exclusion, and host filtering for host‐associated microbiota, both referring to selection (Stagaman, Burns, Guillemin, & Bohannan, 2017). Models for quantifying the stochastic (Burns et al., 2016; Harris et al., 2017; Ofiţeru et al., 2010; Sloan et al., 2006) and deterministic (Morrison‐Whittle & Goddard, 2015; O’Dwyer, Kembel, & Sharpton, 2015; Stegen et al., 2012; Yeh et al., 2015) processes in different types of microbial ecosystems continue to provide new comprehensive insights regarding the forces governing microbiome assembly. Stochastic and deterministic models are also developed in a subbranch of food microbiology, predictive microbiology. However, these models focus on population dynamics of pathogens or spoilage organisms and aim to quantify intrinsic and extrinsic effects and processing factors that influence them in food products (McMeekin et al., 2008), including fish (Tsironi, Houhoula, & Taoukis, 2020). First generations of predictive microbiology models were deterministic, modelling the impact of abiotic factors (Koutsoumanis & Aspridou, 2017). The field subsequently turned to stochastic models, acknowledging the effect of individual cell behaviour and biotic factors such as the growth of competitors (Mejlholm & Dalgaard, 2015; Valenti et al., 2013).

In fish, many studies of host‐associated ecosystems, focusing either on ontogeny under neutral conditions or on case–control comparisons, reveal ambiguities regarding the relative influence of neutral and non‐neutral processes on microbiota ontogeny: for instance, there were contradictory results between zebrafish and other fish species: in zebrafish, neutral processes generated substantial variation in gut microbiota composition among hosts, but non‐neutral processes (i.e. microbe–microbe interactions, active dispersal or host filtering) increased along host development (Burns et al., 2016). In contrast, in three aquaculture fish species, deterministic processes shaping gut microbiota assembly were mainly at play during the first developmental stages before gradually reducing (Yan et al., 2016). Similarly, in sturgeon, deterministic processes were observed to overwhelm neutral processes during the early gut microbiota assembly (Abdul Razak & Scribner, 2020). More importantly, there were substantial differences when comparing wild and captive populations of the same fish species: for Atlantic salmon, it came out that wild individual's microbiota experienced an increasing role of deterministic factors along development, resulting from host filtering, whereas microbial assembly in farmed individuals was mainly driven by stochastic processes (Heys et al., 2020). This observation has to be paralleled with microbiota diversity contrasts detected between captive and wild parrs issued from the same genetic population (Lavoie, Courcelle, Redivo, & Derome, 2018). The immaturity of the gut microbiota detected in captive individuals would reflect the pervasive effects of neutral processes in an environment characterized by relaxed selective pressures as observed in aquaculture rearing systems. On the contrary, wild parr microbiota was much more structured with significantly higher disparity and lower richness, which would reflect the action of deterministic processes. Therefore, the interplay between the underlying evolutionary processes governing microbiota ontogeny in farmed and wild fish populations is still poorly understood. In aquaculture facilities, microbial conditions differ considerably from those in natural environments. In pristine wild environments, waters are often oligotrophic, thus maintaining microbial loads and community composition relatively stable. In aquaculture rearing systems, bacteria and other microbes are continuously introduced to the rearing environment through intake water, feed and fish excrement, thus exerting noticeable temporal variations in microbial loads and community composition. In addition, a common goal in aquaculture is to lower as much as possible microbial loads, and thus, chemical disinfection is a standard water management tool. However, disinfected water typically contains dysbiosed microbial communities (Attramadal et al., 2012). Those will benefit from an environment with high‐carrying capacity, with a variable supply of organic matter (faeces, food, dead biomass). These conditions favour fast‐growing generalist populations (i.e. opportunistic microbes), which in turn favour disease outbreaks (De Schryver & Vadstein, 2014). Because finfish are in direct contact with the rearing water microbial community, their microbiota is highly dependent on bacterioplankton and food‐associated microbes, even more so in recirculating water systems. In turn, fish faeces microbiota will interact with bacterioplankton (De Schryver & Vadstein, 2014). Therefore, a combination of high loads of organic material (residual food, faeces), and unstable environmental conditions in aquaculture systems (sporadic disinfection, cleaning of ponds), will favour dominance of non‐neutral processes in microbial community assembly, favouring generalist and opportunistic microbial strains. Such microbial community shift is thus making the control of disease outbreaks very challenging. Under the lens of community ecology, the impairment of host physiology is now increasingly interpreted in terms of fitness equilibrium disruption among microbiota members, translating in turn to a shift from immunological equilibrium to inflammation/disease (Costello, Stagaman, Dethlefsen, Bohannan, & Relman, 2012). This equilibrium disruption results from host filtering failure via immune response impairment and changes in terms of competitive exclusion patterns. Consequently, community ecology should certainly be considered to sustainably manage microbial homeostasis in aquaculture environments.

## 4 CHANGES IN THE SELECTIVE CONTEXT ALONG THE PRODUCTION CHAIN

From the eggs to the consumed products, fish harbour complex microbial communities shaped by the combined ontogeny/contamination and various biotic and abiotic factors. Those factors were documented to exert both neutral and non‐neutral evolutionary forces controlling the establishment of microbial symbionts during the life of a fish (Burns et al., 2016; Cheaib et al., 2020) and spoilage‐associated microbial communities in food (Boziaris & Parlapani, 2017; Gram & Huss, 1996). Although there are numerous works documenting the impact of abiotic factors (i.e. environmental) such as nutrients, xenobiotics, temperature, pH, salinity, and others, on different microbial communities, both aquatic and terrestrial, either free‐living or host‐associated (Díaz‐Sánchez et al., 2018; Lladó, López‐Mondéjar, & Baldrian, 2018; Logares et al., 2020; Zhao et al., 2018), biotic factors, which include interactions between host and associated living communities (either microbial or parasitic), are much less documented. The same can be said for the impact of abiotic and biotic factors on food microbial communities. While the importance of biotic factors, that is microbial interactions in spoilage development, has been acknowledged for decades (Gram et al., 2002), the mechanisms by which microbial interactions influence whole community assembly in fresh food are only beginning to be addressed. The following section summarizes our current understanding of these parameters on fish‐associated microbial communities, first for abiotic factors and then for biotic factors considering host–microbiota, parasite–microbiota and microbial interactions.

### 4.1 Abiotic factors

Among abiotic factors (i.e. environmental), experimental variations in water physico‐chemistry were observed to drive microbial dysbiosis in fish, potentially modifying functional repertories (Kokou et al., 2018; Sáenz et al., 2019). Interestingly, dysbiosis is tissue‐specific: external microbiome such as skin bacterial communities was observed to shift at the same time than the bacterioplankton, whereas gut bacterial community shift occurred at a later stage of xenobiotic exposure, suggesting that gut responded mainly to the delayed host physiology disturbance rather than the permanent exposure of its body with the surrounding xenobiotic‐contaminated water (Cheaib et al., 2020). Similarly, in tambaqui (*Colossoma macropomum*), an Amazonian fish tolerant to pH variation, faecal and skin microbiota exhibited different patterns following acclimation to acidic water. Skin microbiota was still dysbiosed, whereas the faecal microbiota converged with control the group, thus suggesting a stronger resilience capacity of the intestinal microbiota than cutaneous microbiota (Sylvain et al., 2016). Temperature variation was extensively documented to affect poikilothermic vertebrates, including fish (Bletz et al., 2017; Longo & Zamudio, 2017; Makarieva, Gorshkov, & Li, 2005) in terms of developmental, metabolic and physiological processes (Patterson, Mims, & Wright, 2013; Réalis‐Doyelle, Pasquet, De Charleroy, Fontaine, & Teletchea, 2016; Schulte, Healy, & Fangue, 2011) and in turn microbiota composition (Kokou et al., 2018; Kueneman et al., 2019). In aquaculture, temperatures above the optimal growth temperature are documented to induce stress, to reduce growth (Austin & Austin, 2012, 2016) and to disrupt microbial communities in the fish gut (Huyben et al., 2018). Several studies stated that changes in water temperature influence the load, virulence and diversity of gut microbes in salmonids (Neuman et al., 2016; Waché et al., 2006). In tilapia, cold exposure drove dramatic changes in gut microbiome composition, by increasing *Proteobacteria* (*Vibrionales* and *Alteromonadales*) and decreasing all other phyla (Kokou et al., 2018). Acclimation to water salinity also induced compositional shifts in fish microbiota. Clear differences were detected in anadromous species such as Atlantic salmon between returning adults (freshwater) and adults sampled in marine water. Noticeably, returning adults gut microbiota were highly similar to that of adults sampled in the marine environment (Llewellyn et al., 2016). In the aquaculture context, the transition from freshwater to saltwater was observed to induce a shift in the skin mucus microbiota, both in terms of richness and evenness (Lokesh & Kiron, 2016). This compositional shift mainly resulted from a strong increase in Proteobacteria, whereas Bacteroidetes, Actinobacteria, Firmicutes, Cyanobacteria and Verrucomicrobia decreased. In gut microbiota, transition from freshwater to saltwater resulted in different compositional changes: *Firmicutes* increased, whereas both *Actinobacteria* and *Proteobacteria* decreased (Rudi et al., 2018). Interestingly, by conducting experimental variations of water salinity, Schmidt, Smith, Melvin, and Amaral‐Zettler (2015) showed that deterministic processes have mainly driven fish microbiome assembly along acclimation to water salinity. The osmotic pressure of environmental water is pivotal in the early process of microbial recruitment. Hypo‐osmolarity was demonstrated to modulate innate immunity in skin keratinocytes of newly hatched zebrafish embryos (Galindo‐Villegas et al. 2016), which in turn will exert control of early microbial symbionts recruitment (Galindo‐Villegas, García‐Moreno, de Oliveira, Meseguer, & Mulero, 2012). This original study stresses the need to tightly monitor water chemistry in hatcheries to secure fish larvae survival (Lee & Krishnan, 1985).

Anthropogenic stressors such as xenobiotics were observed to decrease microbial diversity, both in terms of taxonomy and functional repertoires. Cheaib, Le Boulch, Mercier, and Derome (2018) highlighted adaptive signatures in a lake metacommunity system along a polymetallic pollution gradient. Principally, they detected a signature of taxon‐function decoupling in bacterioplankton of moderately and highly polluted lakes, and a gradual deterioration of essential ecological functions such as photosynthesis and secondary metabolism in bacterioplankton from highly polluted lakes. In the aquaculture industry, the main xenobiotic faced by microbial communities is antibiotics, which are mostly used as a therapeutic tool (Austin & Austin, 2016; but see Cabello, 2006). Although antibiotic curative use has been associated with reduced pathogen infections, recent studies highlighted deleterious effects for fish microbiota. In Atlantic salmon (*Salmo salar*), curative doses of florfenicol and oxolinic acid triggered gut adherent community shifts, notably by altering composition and abundance of dominant bacterial phyla (Gupta, Fernandes, & Kiron, 2019). Furthermore, the impact of florfenicol was tested on the gut microbiome functional repertories using a metagenomic approach in pacu (*Piaractus mesopotamicus*). Noticeably, the relative abundance of both antibiotic resistance genes (ARGs) and mobile genetic elements (MGEs) significantly increased during the antibiotic exposure, in addition to plasmids and phage gene prevalence (Sáenz et al., 2019). Because plasmids and phages are vectors of horizontal gene transfers and are generally carrying ARGs and MGEs (Trudel et al., 2016; Vincent et al., 2015), these results suggest that antibiotic administration exerts a noticeable selective pressure in microbial communities in the aquaculture environment. Prophylactic administration has been associated with disease outbreaks in aquaculture (Cabello et al., 2013). The mechanism of microbiota dysbiosis triggered by prophylactic use of antibiotics was validated in zebrafish, where a low dose of olaquindox increased the susceptibility to *Aeromonas hydrophila* infection (He et al., 2017). Last, but not least, nutrition is an important factor driving microbiota assemblage and functional activity, as it directly impacts the host development and physiology (reviewed in Miles & Calder, 2015). This is particularly true for dietary fat which is a rich source of energy upon which depends on normal growth, immune response and in turn disease susceptibility in fish and other vertebrates (Jin et al., 2013). In zebrafish, different dietary fat levels were associated with distinct gut microbiota compositions at different ages. The amount of fat in the diet had distinct age‐specific effects on gut community assembly (Wong et al., 2015). In modern aquaculture, high‐fat diets are commonly used as the main energy source, substituting for costly proteins. However, high‐fat diets were observed to trigger metabolic stress, worsen the effect of antibiotics and ultimately favour disease (Limbu, Ma, Zhang, & Du, 2019). Another issue in fish nutrition in aquaculture is the use of alternative plant‐based protein sources to replace fishmeal in diets for piscivorous species. It is a common practice for farmed Atlantic salmon (Salmo salar) (Krogdahl, Bakke‐Mckellep, Røed, & Baeverfjord, 2000). However, these alternative‐feed ingredients cause symptoms of compromised intestine function. Bacterial groups associated with diet‐induced gut dysfunction are lactic acid bacteria (LAB) (*Weissella*, *Leuconostoc*, *Lactobacillus*, *Pediococcus,* and *Carnobacterium*), with the relative abundance being 18 times higher in the digesta of fish fed vegetal protein than in fishmeal‐fed fish (Gajardo et al., 2017). Although digesta‐associated microbiota showed a clear dependence on the diet composition, mucosa‐associated microbiota appeared to be less affected by diet composition, but exhibited a similar trend regarding the relative abundance of LAB: four times higher in the gut epithelium of fish fed vegetal protein than in fishmeal‐fed fish (Gajardo et al., 2017). Although there is yet no evidence regarding a possible higher influence of biotic factors relative to abiotic in driving microbiota assembly and homeostasis in aquaculture environments, it remains that sustainable management of rearing environments must consider these factors to harness evolutionary forces they induce.

Finally, the above‐mentioned abiotic factors that shape host microbiota, but also condition the properties of the animal flesh such as the protein and fat content, will ultimately contribute to shape the microbial niches in food products. Indeed, in food, abiotic factors impacting microbial development comprise intrinsic properties of the matrix, such as its nutrient composition, water activity, pH and redox potential as well as extrinsic properties such as the atmosphere and temperature in which it is processed and stored. In the case of fish, low temperatures are the most commonly applied hurdle, generally directly after harvesting to inhibit deterioration (Tsironi et al., 2020). Other notable examples include the use of salt, smoke, acids, competitive microorganisms and redox potential modifications (Leroi et al., 2008). Every food preservation method either manipulates abiotic (e.g. temperature, pH, antimicrobial additive), biotic factors (e.g. biopreservation and fermentation) or both. Control and modification of the abiotic factors have been extensively studied in the food industry to implement hurdles to bacterial growth (Leistner, 2000; Leroi et al., 2008). Each of the factors applied within a certain range can influence microbial growth, and the hurdle effect of temperature, water activity, pH, atmospheric modifications and xenobiotics (additives) during fish preservation has been reviewed in Tsironi et al. (2020). Because the goal is to inhibit pathogens and slow the growth and activity of spoilage microorganisms, the studies mostly report counts of targeted bacterial groups. Thus, in fish products how selection takes place once initial contamination has occurred is still not clearly established. However, packaging and the food matrix composition appear to act as important factors. A study by Chaillou et al. (2015) reported that the initial microbiota of fish flesh has a low abundance and a high diversity, and spoiled products have a lower richness than fresh products. Yet, in salmon and cod fillets, the authors report that the selective pressures exerted during storage were weaker than for other food products types of animal origin inducing only a small reduction in richness. Interestingly, microbiota from fish products were shown to have a higher richness than other animal food types. The study also found that the process of washing and smoking strongly affected the contaminants originating from fish gut compared to the contaminants of environmental origin.

### 4.2 Host–microbiota interactions

Genetic diversity within and among host populations was demonstrated to generate variation in immune response, which in turn will contribute to the inclusion of beneficial/neutral microbes and the exclusion of potential pathogens (Gómez & Balcázar, 2008; Hooper, Littman, & Macpherson, 2012). In their pioneering work on Brook char (*Salvelinus fontinalis*), Boutin et al. (2014) provided the first evidence that host genotype influences microbiota taxonomic composition and that specific host genomic regions regulate the recruitment of three specific bacterial genera; *Lysobacter*, *Rheinheimera and Methylobacterium*. Interestingly, these three selected taxa are known for their beneficial antibacterial activity and represent potentially important genera providing protection against pathogens (Boutin et al., 2014).

The hypothesis that host genes associated with the selective recruitment of microbial symbionts, either beneficial or detrimental, are connected with immune functions was tested in fish in both natural and controlled conditions. First, knowing that water temperature is involved in shaping large‐scale patterns of pathogen diversity and virulence, Dionne et al. (2007) validated the general hypothesis that polymorphism at genes of the major histocompatibility complex (MHC) was correlated with the pathogen diversity in salmon rivers. This hypothesis was further tested on Chinook salmon fry kidney sampled in natural rivers where susceptibility associations between a few of the MHC class I and II alleles and specific bacterial parasites were evidenced (Evans & Neff, 2009). Similarly, evidence of a functional relationship between specific MHCII beta alleles and bacterial pathogens in natural conditions was reported in whitefish (Pavey et al., 2013). Second, in the threespine stickleback, MHCII the genotype was hypothesized to contribute to the recognition and regulation of gut microbes, as MHC polymorphism was associated with microbial variation among hosts. Interestingly, hosts with more diverse MHC motifs had less diverse gut microbiota (Bolnick et al., 2014). A more recent study addressing interactions between host genotype and the microbiota revealed subtle signatures on expression patterns of genetic pathways associated with the innate immunity (Small, Milligan‐Myhre, Bassham, Guillemin, & Cresko, 2017). These genotype‐by‐environment interactions may prove to be important to understand the host genetic mechanisms commonly underlying sometimes complex molecular relationships with their resident microbes (Small et al., 2017). The relative contribution of the host genetic background and environmental filtering on the colonization processes have been documented in stickleback and Atlantic salmon natural populations (Lavoie et al., 2018; Smith, Snowberg, Gregory Caporaso, Knight, & Bolnick, 2015). Population‐level differences in stickleback gut microbiota were observed to depend more on host genotype than on transient environmental effects (Smith et al., 2015). More heterozygous populations tended to exhibit lower beta diversity (among‐individual variation) in their gut microbiota. Moreover, natural population stocking of Atlantic salmon with hatchery‐raised parrs issued from population‐specific wild breeder pairs provides a particularly suitable model system to disentangle effects of host genotype and environment on microbiota ontogeny (Lavoie et al., 2018). By comparing individuals belonging to the same population genetic background that were grown either in controlled or natural conditions, this study revealed that the rearing environment played a pervasive role in shaping the gut microbiota taxonomic composition. Indeed, the composition of the gut microbiota of wild individuals was very specific to their own river, yet that of captive individuals, although issued from wild breeder pairs from each of the two river populations, was very specific to the hatchery, with an almost total absence of the genotypic signature of the original population. This comparison of genetically similar fish families in wild and aquaculture environments emphasizes the need to prioritize the management of the rearing microbial community to secure optimal development of the fish.

In addition to the pervasive role played by the rearing environment, host‐specific factors are nonetheless determinant in shaping the fish microbiota along developmental and life stages. During early developmental stages, fish microbiota composition is a highly dynamic system and is strongly correlated with age (Stephens et al., 2016; Trinh, Bakke, & Vadstein, 2017). In wild populations, life‐cycle stages were also observed to define both the diversity and identity of microbial assemblages in the gut, with evidence for community destabilization in migratory phases, as highlighted in a pioneering survey on Atlantic salmon (Llewellyn et al., 2016). In controlled conditions, larval development in Atlantic cod was observed to structure the microbiota, possibly through a change in selection pressure due to host–microbe and microbe–microbe interactions (Trinh et al., 2017). This phenomenon was further studied with the zebrafish model, revealing stage‐specific signatures in the intestinal microbiota, as community shifts were apparent during periods of constant diet and environmental conditions (Stephens et al., 2016). However, the authors also noticed a growing inter‐individual variation along the developmental stages. This unexplained variation across individual hosts, observed in other vertebrate, was addressed under the lens of community ecology in a companion paper (Burns et al., 2016). In this study, Burns et al. (2016) aimed to assess the contribution of neutral and non‐neutral processes in shaping the gut microbiota from larvae to adult by testing whether the observed community assembly process fitted with the predictions of a neutral model, which assumes that community assembly is driven solely by chance and dispersal. Overall, the results showed that the relative importance of non‐neutral processes, such as microbe–microbe interactions, active dispersal or selection by the host, increased as hosts mature (Burns et al., 2016). These observations were further explored in the context of constant and gradual selection regimes exerted by two sublethal cadmium chloride dosages in yellow perch juveniles (Cheaib et al., 2020). In this work, nonlinear least squares models (NLS) suggested that stochasticity mainly drove taxonomic drift in cadmium‐free water communities, whereas host–microbiota assembly evolved in a deterministic (non‐neutral) manner. Furthermore, network analysis detected pervasive negative correlations between taxa in both selection regimes in skin, besides the taxonomic convergence with the environmental bacterial community, suggesting a loss of colonization resistance resulting in the dysbiosis of host‐associated microbiota. Colonizing microbes exert immunostimulatory and/or immunosuppressive effects on both innate and adaptive immune cells, mainly through their display of microbe‐associated molecular patterns (MAMPs) and secretion of metabolites. Those structural or excreted microbial products will stimulate both innate and adaptive immune pathways, which in turn control colonizing microbes (Negi, Das, Pahari, Nadeem, & Agrewala, 2019). For instance, microbiota MAMPs exert innate immunomodulatory effects, which will filter out, or at least control, pathogenic and some opportunistic strains (reviewed in Kelly & Salinas, 2017). In finfish, the maturation of the adaptive immune system occurs along the late developmental stages. In zebrafish, adaptive immunity becomes fully functional between 21 and 28 d.p.f (Lam, Chua, Gong, Lam, & Sin, 2004), which coincided with a shift in gut microbial communities, despite other major driving factors remained stable (diet, water chemistry) (Burns et al., 2016). In a companion study, Stagaman et al. (2017) observed that adaptive immunity was a significant factor in shaping the zebrafish gut microbiota. More specifically, it emphasized individual effect on gut microbiota assembly over the contribution of random microbial symbionts dispersal. This inter‐host variability in microbiota composition may be partially explained by the differences in terms of intestinal immune molecules, as observed for macrophages in adult zebrafish (Earley, Graves, & Shiau, 2018). Investigating how fish larvae would be protected against infection before their adaptive immune system becomes fully matured, and Galindo‐Villegas et al. (2012) showed that early microbial symbionts were mainly recognized through the TLR/MyD88 signalling pathway. These pioneering symbionts were observed to induce up‐regulation of several pro‐inflammatory and antiviral genes. This suggests that innate immune system may play a role in controlling microbiota composition at the host level. At the host population level, however, Burns et al. (2017) observed that the ability of *myd88* in controlling microbiota ontogeny was overwhelmed by the inter‐host dispersal of microbial symbionts. Therefore, in an aquaculture environment, especially during egg incubation and early larval stages, it stresses the necessity to control the rearing environment microbial community. Indeed, the bacterioplankton is known to exert a pervasive influence on fish microbiota assembly (Bakke, Attramadal, Vestrum, & Vadstein, 2019).

### 4.3 Parasite–microbiota interactions

With the global growth in aquaculture production, an increase in parasite outbreaks has been reported in most aquaculture systems (Bui, Oppedal, Sievers, & Dempster, 2019). Transdomain interactions between eukaryotic parasites (helminths, protozoa and fungi) and the bacterial microbiota can critically alter the immune landscape of the gut (Gause & Maizels, 2016; Giacomin, Croese, Krause, Loukas, & Cantacessi, 2015), thus affecting the overall host health status, either driving or protecting against dysbiosis and inflammatory diseases. For instance, in Atlantic salmon a copepod parasite is known to trigger secondary bacterial infections. To this respect, network analysis of microbial taxa of infected fish revealed that skin microbiota associated with high louse burdens was characterized with an increase of connections between multiple pathogenic genera (*Vibrio*, *Flavobacterium*, *Tenacibaculum*, *Pseudomonas*) (Llewellyn et al., 2017). The decrease of skin commensals and increase of opportunistic bacteria were also observed in rainbow trout, where parasite infection led to the loss of *Proteobacteria* in favour of members of the *Bacteroidetes* phylum (Zhang et al., 2018). Parasite–microbiota interaction is bidirectional as the microbiota can alter a parasite's colonization success, replication and virulence, shifting it along the parasitism–mutualism spectrum. An experimental infestation in zebrafish showed that parasite exposure, burden and intestinal lesions were correlated with gut microbial diversity. In this study, using a machine learning approach, gut microbiota phylotypes were associated with parasite success (Gaulke et al., 2019). Mechanisms and consequences of parasite–microbiota interactions are therefore starting to be elucidated. Overall, gut colonizing organisms exist along a wide interaction spectrum from parasitism to mutualism, which may change according to the physiological status of the host organism (Méthot & Alizon, 2014). For instance, many parasitic eukaryotes only cause disease sporadically, while many host organisms remain asymptomatic (Parfrey, Walters, & Knight, 2011; Wammes, Mpairwe, Elliott, & Yazdanbakhsh, 2014). Furthermore, some of them were observed as beneficial for their host, as exemplified with cestode parasitism of brine shrimp, which enhanced host resistance to a xenobiotic by promoting antioxidant synthesis (Sánchez et al., 2016). Similarly, many gut microbes can be considered opportunistic pathogens, in that they do not ordinarily cause harm, but are capable of causing disease when the host encounters a physiological stress (Boutin, Bernatchez, Audet, & Derome, 2013). Therefore, there is growing evidence that interactions between gut microbes and parasites can influence each other's pathogenicity, making them a great concern in aquaculture. Moreover, the presence of parasites in farmed fish causes concern not only for fish health, but also for food safety (Lima dos Santos & Howgate, 2011) as well as quality, as they can act as bacterial vector when migrating from the intestine to the muscle. Some infections by parasitic worms can lead to an increased load of bacteria in fish flesh, including SSO (Svanevik et al., 2013). Given the role that these parasites can play in shaping the microbial communities associated with farmed fish, parasite–microbiota interactions may deserve more attention.

### 4.4 Microbial interactions

Recent advances in microbial ecology have highlighted the importance of microbial interactions in multiple contexts, but our understanding of their mechanisms and their role in evolutionary processes is still in their infancy (Layeghifard, Hwang, & Guttman, 2017). Understanding microbial interactions is essential to understand how microbial communities function and whether it is possible to manipulate their dynamics to maintain health (Bentzon‐Tilia, Sonnenschein, & Gram, 2016) or postpone spoilage in foods (Andreevskaya et al., 2018). Although caution in the interpretation of specific interactions is warranted in correlation‐based microbial association network analyses, valuable information can be gathered, especially about niche requirements of species in an ecosystem (Carr, Diener, Baliga, & Gibbons, 2019) or about the impact of treatments administered to a host or product on the resulting network topology.

The importance of microbial interactions in the fish host has been recognized in several studies, either in stress trials (Boutin et al., 2013; Chen et al., 2018; Gupta, Fernandes, et al., 2019; Llewellyn et al., 2017; Sylvain et al., 2016) or in natural conditions (Sylvain et al., 2019), whereas this approach is still scarce for food spoilage (Chaillou et al., 2015; Zotta, Parente, Ianniello, De Filippis, & Ricciardi, 2019). For instance, Boutin et al. (2013) showed for the first time that interactions among bacterial genera associated with known probionts (*Sphingomonas*, *Methylobacterium*, *Propionibacterium* and *Thiobacter*) were suppressed following stress exposure in brook char, whereas new interactions between strains deemed as opportunistic pathogens in salmonids (*Aeromonas salmonicida*, *Acinetobacter* spp., *Pseudomonas chlororaphis*, *Psychrobacter immobilis*) appeared. In Atlantic salmon, Gupta, Fernandes, et al. (2019) reported that stress exerted by two antibiotics altered the inferred gut microbial interaction network differentially in terms of connectivity, betweenness and hub degree. Chen et al. (2018) exposed zebrafish to a xenobiotic (polybrominated diphenyl ether DE‐71). Gut microbiota of individuals exposed to DE‐71 exhibited a correlated increase of 13 bacterial genera (*Streptococcus*, *Staphylococcus*, *Moraxella*, *Mycobacterium*, *Haemophilus*, *Bacillus*, *Fischerella*, *Aeromonas*, *Pseudomonas*, *Listeria*, *Helicobacter*, *Neisseria* and *Leptospira*), all of which are negatively correlated with other rare genera. Collectively, these studies demonstrate that exposure to different types of stress selectively promotes a rewiring of the microbiota. However, it is not clear to what extent this community change translates into adaptation to the impaired physiological conditions, or simply results from a dysbiosis state. Indeed, the 13 aforementioned genera in Chen et al. (2018) were either positively or negatively associated with beneficial functions or phenotypes. Nevertheless, microbial communities are exposed to continuously changing selection pressures, subjected to a never‐ending ecological succession process (Curtis & Sloan, 2004). These compositional dynamics may result either from strain assortment, cross‐feeding or thanks to functional redundancy, or from selecting alternative strains towards securing any given function of interest. For instance, it has been shown that dynamic microbial communities can provide functional stability (Cabrol & Malhautier, 2011; Escalas et al., 2017).

In foods, using co‐occurrence patterns, Chaillou et al. (2015) showed that microbial community structure shifts between fresh and spoiled fish. Zotta et al. (2019) performed network analysis on bacterial metabarcoding data of Hake and Plaice fillets. They concluded that habitat filtering may dominate the interactions that they report and confirm that spoilage microorganisms are core members and negative hubs. Moreover, their network is comparable to various other food biomes (Parente et al., 2018). To our knowledge, no studies have directly compared microbial association networks in live fish microbiota and the subsequent food products. However, Parente et al. (2018) performed a metanalysis comparing food, their host and soil microbial association networks. They found that the proportion of positive interactions was similar between food and host, but the food networks were smaller, with a higher density, a lower average path length and a lower clustering coefficient. Properties of microbial association networks in food were closer to those of the host than to soil in their dataset. Although they shared similarities with host microbial association networks, food microbial communities lacked the scale‐free structure observed in environmental samples and many other biological networks. Fermented and spoiled food had simplified microbial association networks dominated by negative hubs.

The drastic contrasting conditions from the previous aquatic environment are likely to select for r‐strategist microorganisms rather than k‐strategist (Box 2). Indeed, in some populations, a trade‐off can be shown in a selective environment between growth rate and yield (Filteau, Charron, & Landry, 2017; Ibstedt et al., 2015). Carrying capacity may be optimized within hosts where nutrients may be scarce, but on a rich substrate such as fish flesh, strains with an optimal growth rate would be favoured. Therefore, a switch in these two types of selection regime can be assumed. The resulting microbial community networks are likely to present different structures, although they may share members.

#### BOX 2 Microbial community modulation strategies

**Probiotics**. In aquaculture, the definition was adapted by Merrifield et al. (2010) who defined a probiotic as: “a live, dead or component of a microbial cell that, when administered via the feed or to the rearing water, benefits the host by improving either disease resistance, health status, growth performance, feed utilization, stress response, which is achieved at least in part via improving the hosts or the environmental microbial balance.” Some authors also define dead probiotics as **paraprobiotics** (Choudhury & Kamilya, 2018). Probiotics can also be used in combinations and a connected concept, **competitive exclusion culture,** that is a mixture of microorganisms derived from a healthy host that are maintained to exclude pathogens, is a promising avenue of research (Melo‐Bolívar et al., 2019). This review focuses on live strain administration.

**Prebiotics**. A prebiotic is defined by Roberfroid (2007) as: “a nondigestible food ingredient that beneficially affects the host by selectively stimulating the growth and/or activity of one or a limited number of bacteria in the colon, that can improve the host health.” A prebiotic dietary ingredient follows three main criteria: (1) resist gastric acidity, hydrolysis by digestive enzymes and gastrointestinal absorption; (2) be fermented by the intestinal microbiota and (3) be able to selectively stimulate the growth and activity of beneficial bacteria (Gibson, Probert, Loo, Rastall, & Roberfroid, 2004).

**Synbiotics** refer to nutritional complements combining probiotics and prebiotics (Cerezuela, Meseguer, & Esteban, 2011). Synbiotics aim to simultaneously seed and maintain probiotic strains as the dominant species in the gut after treatment (Rurangwa et al., 2009).

**Quorum quenching** is the process of disrupting quorum sensing, that is a mode of social life communication among microbes dependent on cell density that regulates a plethora of behaviours, including virulence in many aquatic pathogens such as *Aeromonas*, *Vibrio* and *Edwardsiella* (Chu & McLean, 2016). **Quorum sensing inhibitors** are synthetic or natural molecules that interfere and disrupt this communication mechanism.

**Bioprotective cultures** are microorganisms that show antimicrobial activity that inhibit either spoilage microorganisms or pathogens. Bioprotection involves adding specific cultures, that is live microorganisms to foods, to control its microbiological status without changing its technological and organoleptic properties (Ben Said et al., 2019). Bioprotection effects may include the production of organic acid, bacteriocins or other antimicrobials, competition for nutrients, cell‐to‐cell contact and niche effects (Cifuentes Bachmann & Leroy, 2015).

**Bacteriocins** are antimicrobial compounds of a peptidic nature that exert either bacteriostatic or bactericidal effects, usually on similar or closely related bacteria. In the food sector, nisin is used for the preservation of smoked fish.

**Phage therapy**. Bacteriophages, or phages for short, are viruses that infect bacteria with high (species) to very high (strain) degree of specificity. Phage therapy is the use of bacteriophages to treat pathogenic bacterial infections. Lytic phage, those that lyse bacterial cells immediately after replication are better suited for therapy applications than temperate phages that integrate the bacterial host genome and eventually trigger lysis depending on host status (e.g. acute stress).

**Fermentation** is a traditional food preservation method typically involving product acidification and often performed by lactic acid bacteria. In fish, this process is mostly used in Asia (Zang, Xu, Xia, & Regenstein, 2020).

**Biofilters r/K**

This holistic strategy relies on the ecological theory of r/K selection (MacArthur & Wilson, 1967), which hypothesize that stable environmental conditions will favour over slow‐growing specialists (**K‐strategists**) over fast‐growing opportunists (**r‐strategists**). Biofilter r/K aims therefore optimizing fish microbiota ontogeny by enriching environmental microbial communities with K‐strategists. The principle of this approach is to stabilize the water at low microbial carrying capacity, by lowering resources and maximizing their use by K‐strategists. This can be obtained by using a recirculating aquaculture systems (RAS), which contains biofilters (for the conversion of toxic ammonia to nitrate) with a large surface area for bacteria, and the microbial carrying capacity is relatively stable throughout the system. The biofilter K/r approach was pioneered by Olav Vadstein for cod (*Gadus morua*) in the 1990s.

The changes in selective context along the production chain shaping seafood bacterial community diversity are still poorly understood given the wide variability of consumed species and transformation processes. Moreover, how members of these microbial communities compete or interact with each other merits further attention as these could have important consequences for applications modulating fish product microbiota. For instance, controlling the total contamination levels is of paramount importance in food preservation and may be achievable by targeting specific keystone species in the microbiota since it has recently been shown in a model microbiota that deletion of specific species can lead to a reduction of biomass or reduce growth rate (Gutiérrez & Garrido, 2019). However, a biomass reduction does not necessarily mean a spoilage or pathogen reduction. Nevertheless, identifying and targeting keystone species or gatekeepers (Dai, Chen, & Xiong, 2019) in microbial communities is a promising approach to regulate microbial community behaviour. Furthermore, incorporating biotic effects into these models, such as interactions between groups of microorganisms, would help improve our mechanistic understanding of microbial interactions on food spoilage and further develop modulation strategies.

## 5 MICROBIAL COMMUNITY MODULATION STRATEGIES FROM EGG TO FORK: POTENTIAL FOR RISK, CONFLICT AND OPPORTUNITIES

**Figure 1:**
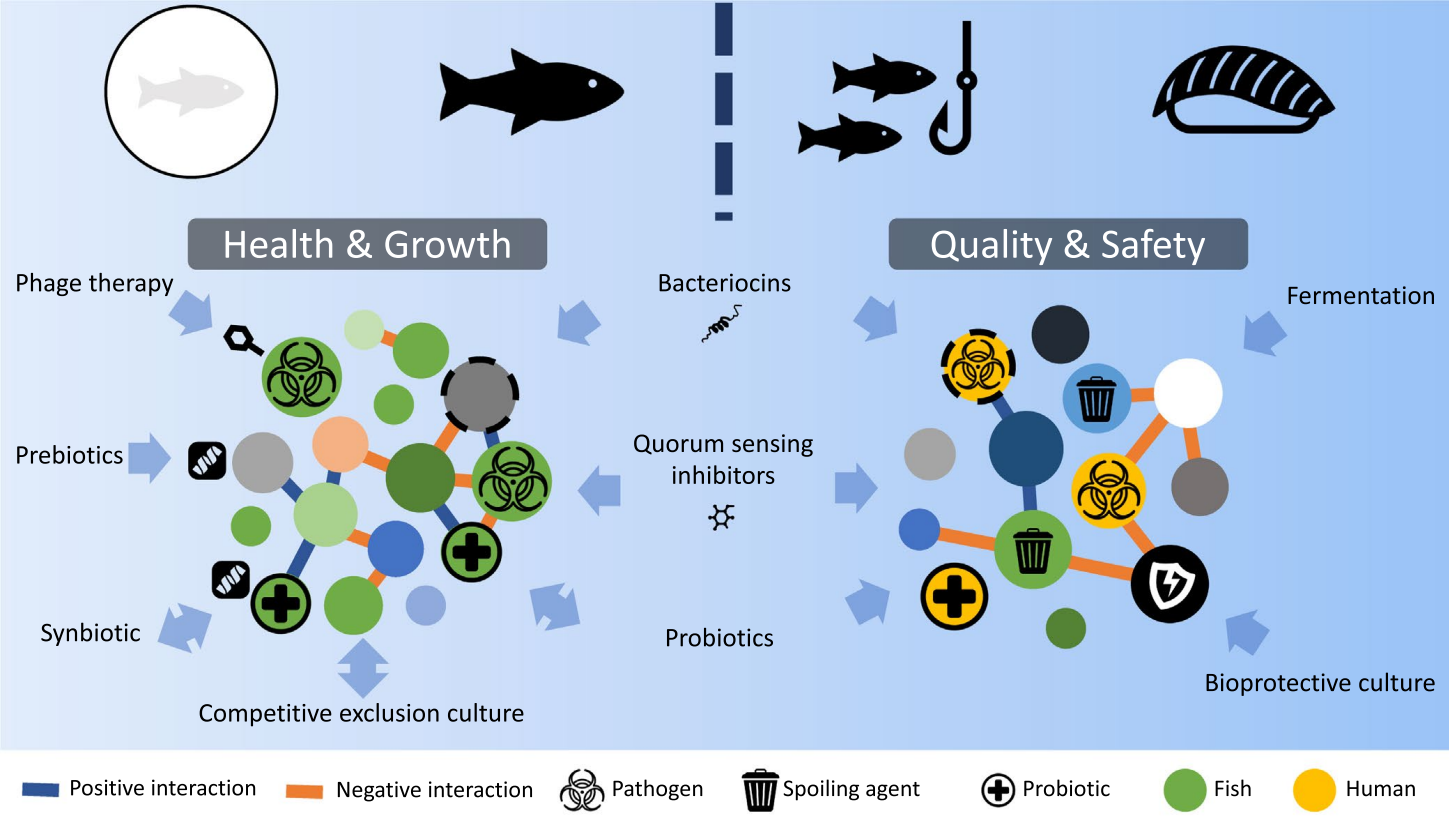
Microbiota modulation applications in aquaculture and food transformation. While objectives differ between the two sectors, aquaculture (left) aiming to control fish pathogens and food transformation (right) aiming to control spoiling agents and human pathogens, the principles that govern microbial community ecology are similar. Microbiota of fish host and fish product are both a dynamic assemblage of microorganisms (circles) shaped by abiotic and biotic forces, including positive (blue line) and negative (orange line) interactions among them. Association to fish (green) or human (yellow) is denoted by circle colour, and functional role (pathogen, spoiling agent, probiotic, etc.) is depicted by symbols. LAB, lactic acid bacteria

As the microbial communities of fish hosts and food products are being increasingly characterized and the factors governing their assembly and dynamics are being further understood, opportunities to shape their dynamics and functions undoubtedly arise. Multiple microbial community manipulation strategies are being investigated and implemented to meet with current challenges in aquaculture and the food industry (Figure 1, Box 2). Fish production for human consumption involves a continuum of microbial communities that respond to their local biotic and abiotic context. This directed continuum brings challenges to consider when developing applications, but also offers opportunities to bridge pre‐ and postmortem applications.

In the aquaculture industry, various curative and prophylactic alternative approaches were developed in the last half‐century, aiming to decrease the use or even get rid of the dependence on antibiotics. As an invariant molecule, the selective pressure of an antibiotic treatment is constant and directional in such a way that the emergence and selection of resistance mechanisms in targeted microbes are favoured, thus rendering the antibiotic treatment no longer effective. In addition, the generalized use of antibiotics will translate into the release of molecules into the environment, which in turn will further favour the selection of resistance genes in various natural microbial communities (Cabello, Godfrey, Buschmann, & Dölz, 2016; Cabello et al., 2013; Martinez, 2009). On the contrary, using live organism as biocontrol agents poses less risk of resistance development, as the living organism will benefit from its phenotypic plasticity to exert antagonism vis‐à‐vis the targeted pathogenic strain and, if new strains are isolated on a regular basis on healthy host organisms, have the possibility to co‐evolve with the pathogen. This arm race mechanism stands not only for probiotics, but also for phage therapy, as targeted bacterial pathogens can become resistant to their phages. In response, phages can mutate and therefore evolve to counter phage‐resistant bacteria, thus stressing the necessity to isolate phage mutant that counter phage‐resistant bacteria regularly (Matsuzaki et al., 2005). This can be achieved using in vitro trials to select efficient phage mutants (Uchiyama et al., 2011). Moreover, because of the high mutation rate of lytic bacteriophages (e.g. 10^−6^µ/generation), lytic polymorphism is theoretically possible following phage administration, thus modifying specificity and/or efficiency of the treatment.

These considerations highlight the fact that harnessing a complex system such as a microbial community is a challenging task as the members have various interacting relationships such as cross‐feeding, competitive exclusion, most of which interact with the host immune system, either by promoting or mitigating inflammation, and consequently disease resistance. Therefore, the long‐term use of invariant (quorum sensing inhibitors or prebiotics) or variant (single or multiple probiotic strains, lytic phages) microbiota‐harnessing tools can either favour or disfavour disease resistance. For instance, probiotic being a living organism, its claimed beneficial properties can either result from a constitutive or an inducible mechanism. The latter case being tightly dependent on biotic and abiotic factors, including the surrounding microbial community, the probiotic strain may become less efficient or even harmful in certain conditions. According to FAO guidelines for probiotic use, based on Shanahan (2012), “The adverse effects and the severity of the effects of a probiotic could be context specific and depend on the susceptibility (immunity) and physiological state of the host.” To this respect, the relevance of using the probiotic approach has been questioned for young larval stages (i.e. with immature immune system), as a probiotic strain may trigger a dysbiosis in the microbial community and in turn favour opportunistic strains (r‐strategists) while altering pathogen resistance in larvae microbiota (De Schryver & Vadstein, 2014). Moreover, risks associated with the use of probiotics include the possibility of horizontal transfers of antibiotic resistance to other pathogenic microorganisms, and production of metabolites (e.g. enterotoxins) that may be toxic to the host. In addition, a probiotic strain isolated in one host organism may be toxic when administered to an alternative host, due to the different priority effects (i.e. order and timing of microbial strain colonization determining how species affect one another) that were orchestrating the strain assortment. Therefore, whatever the probiotic effect is—either inducible or constitutive—an allochthonous probiotic strain may trigger deleterious health effects due to negative interactions with microbial residents in the alternative host organism. This phenomenon may theoretically apply for other microbiota managing tools. In phage therapy, for instance, the high specificity to their bacterial host is theoretically preventing any collateral damage on microbiota. In aquaculture, the main claimed advantage of phages is that they might kill specifically planktonic pathogens living in the surrounding water in addition to pathogens proliferating in carrier fish without altering the aquatic bacterial ecosystem (O'Sullivan, Bolton, McAuliffe, & Coffey, 2019). The lytic spectrum of phages is known to range from one strain to a few bacterial species (Hyman & Abedon, 2010) in such a way that a given phage strain could co‐infect closely related bacterial hosts that are key microbiota members. It can be even worse if a mixture of phages is administered to circumvent resistance development in the targeted bacterial host, as it increases the host range (Chan & Abedon, 2012). Another potential drawback of phage therapy is the possible transduction of virulence factors between bacterial hosts, as well as the fact that the vertebrate host could mount an immune response against the phage itself (Khan Mirzaei et al., 2016).

The safety of phage therapy was assessed in aquaculture (reviewed in Dy, Rigano, & Fineran, 2018). According to the LIFE13 ENVIPHAGE project, phage therapy does neither stimulate the fish immune system nor alter the aquatic bacterial ecosystem (http://www.enviphage.eu). Once again, further analyses on metagenomic data are needed to state whether single strain or phage cocktails may alter functional interactions in both environmental and host‐associated bacterial communities.

Another risk to consider with the use of live organisms is toxicity even though examples of probiotic toxicity are scarcely reported in the literature. A case was reported for a commercial probiotic strain (*Bacillus cereus*) administered to both human and porcine epithelial cells (Trapecar et al., 2011). In fish, a *Micrococcus luteus* strain was identified as the causative agent of disease outbreaks in rainbow trout and brown trout (Pękala et al., 2018), whereas another strain of *M. luteus*, isolated from the gonads and intestines of Nile tilapia (*Oreochromis niloticus*), showed probiotic properties in vivo (Abd El‐Rhman, Khattab, & Shalaby, 2009). In that case, we cannot rule out the possibility these two strains have different properties due to their different host, in addition to the effect of two different experimental contexts. Metagenomic investigations are necessary to figure out to which extent interactions with other members of the resident microbiota are different in prophylaxis and curative use. Taken together, these observations show that the risk assessment of a probiotic strain is a challenging task, stressing that the claimed beneficial properties of a strain critically depend on the conditions of use. This statement stands for any therapeutic tool.

When conducting the risk assessment of a new probiotic candidate, tests are conducted to verify the absence of any transferable antibiotic resistance or virulence genes, toxin production or hyperstimulation of the immune system. Of course, probiotic strains should not be associated with any disease, including zoonosis. Shotgun metagenomics is useful to test how the administration of a given probiotic strain affects the overall microbiota gene pool, and whether functional repertories changes are either associated to promoting or mitigating inflammation. Metatranscriptomics, or dual RNA‐Seq, is even more suitable as it allows integrating both microbiota and host gene expression, including immune‐related genes. Hence, both metagenomics and metatranscriptomics provide valuable tools to model the effect of any microbiota‐harnessing tool, either variant or invariant, on a complex interacting host–microbiota system. In practice, due to the case‐by‐case analysis requested for a reliable risk assessment, including various contexts of use, the challenge will be to identify experimental conditions that are the most relevant to focus on using metagenomics or metatranscriptomics approaches.

Despite the advantages of probiotics, the viability of live bacteria during large‐scale production of feed and during transition through the gastrointestinal tract is not always achieved (Ringø, Dimitroglou, Hoseinifar, & Davies, 2014). To cope with the viability concern of some probiotics, prebiotics have been supplied in fish (reviewed in Ringø et al., 2010) either combined with probiotics as synbiotics (Huynh et al., 2017) or alone to promote the gut microbiota activity. Indeed, prebiotics were observed in fish models to exert a control on the microbiota taxonomic composition (Forsatkar, Nematollahi, Rafiee, Farahmand, & Lawrence, 2017). In general, treatments of fish with prebiotics and synbiotics have beneficial effects on immunological responses, survival, growth, intestinal absorption and health status (reviewed in Hoseinifar, Van Doan, Dadar, Ringø, & Harikrishnan, 2019). To this respect, the prebiotic strategy may be viewed as a positive managing tool as it does not directly impact negatively a single or few strains, but rather promote the activity of beneficial strains.

In food, microbial community manipulation strategies using live microorganisms such as the addition of competitive cultures are very attractive natural hurdles to use in combination with other preservation methods. Fermentation is a famous example of competitive culture and a classical preservation method, still used in many traditional fish preparations around the world (Tanasupawat & Visessanguan, 2013). However, as it modifies the organoleptic properties, it is not a widely used approach for fish preservation, representing less than 12% of human consumption (FAO, 2018). Aside from fermentation, some attempts to manipulate microbial communities with bioprotective cultures in fish products have been successful. Examples of commercial biopreservation application of lactic acid bacteria include the use of *Carnobacterium divergens* M35 in cold‐smoked salmon, *Lactobacillus pentosus* in fresh salmon, and *L. casei* and *L. plantarum* in vacuum‐packed cold‐smoked salmon (reviewed in Ben Said et al., 2019). Nevertheless, developing applications such as bioprotective cultures involves tedious work, mostly based on years of empirical evidence. How individual species or strains could affect the stability and performance of complex microbial communities is still not well known (Gutiérrez & Garrido, 2019). Indeed, bioprotection often involves strains that exert inhibition of specific pathogens, but the impact on the whole microbial community and the exact ecological mechanisms involved is often not well understood. For instance, Wiernasz et al., 2020 documented the effect of six LAB strains using a polyphasic approach that included the characterization of the microbial communities by 16S rRNA gene amplicon sequencing. They observed that both competitive and noncompetitive behaviours could lead to desirable outcomes for biopreservation application. It is clear that technology and variability in microbial interactions and adaptation have to be considered when developing bioprotective applications. A universal bioprotective solution may be difficult to achieve considering the specificities of antimicrobial production and activity. The microbial community compositions may shift over time, requiring continuous attention.

Probiotic and bioprotective strains often possess a molecular antimicrobial arsenal relevant to their environment of origin (Ghanei‐Motlagh et al., 2019). Given the complexity of live cultures, an alternative option is to directly use the molecular compounds. Some authors advocate the search for these invariant microbiota‐harnessing tools as alternative antimicrobials, like quorum sensing inhibitors (Chu & McLean, 2016) and bacteriocins (Sahoo et al., 2016). In food, compounds that underlie spoilage are often under the control of quorum sensing mechanisms. Therefore, the use of QS inhibitors to modulate microbial activity has been investigated, but so far hurdles like instability or toxicity issues have not been overcome (Tiwari et al., 2016). Applications of bacteriocin have been more successful and offer the advantage that their characteristics and mode of action have been well studied, but there is still a vast knowledge gap concerning the evolution of resistance to these compounds which is frequently reported (Kumariya et al., 2019; Willing et al., 2018). There are multiple evolutionary factors shaping the evolution of bacteriocin and other antimicrobial compounds. Therefore, the evolutionary outcome of a large‐scale application of these compounds in aquaculture is hard, if not impossible to predict at this point. One factor shaping bacteriocin evolution is environmental structure. Majeed, Lampert, Ghazaryan, and Gillor (2013) showed that the environment structure drives the outcome of the competition between producers of potent and weak bacteriocins: an unstructured environment with global interactions selectively favours weak producers, whereas a structured environment with local interactions allows coexistence. While the aquatic environment of fish could be considered an unstructured environment, the fish as a host and fish flesh are structured environments. Moreover, bacteriocin could play an important role in maintaining intra‐ and inter‐specific diversity patterns on a local scale in a structured environment (Hawlena, Bashey, & Lively, 2012). Another point to consider is that bacteriocin‐encoding operons can be carried by plasmid or MGEs (Kumariya et al., 2019). A study showed that the unique ecology and evolution of plasmid‐encoded bacteriocins could be explained by their selfish genetic element nature that promotes their own transmission in the population (Inglis, Bayramoglu, Gillor, & Ackermann, 2013). Thus, the application of bacteriocin for pathogen control may have a greater chance of resistance development in aquaculture since this environment is a hotspot for gene transfer (Watts et al., 2017). Most bacteriocins have a narrow inhibition spectrum, mostly inhibiting close relatives, while other compounds can have a broader spectrum, for instance nisin can inhibit numerous Gram‐positive bacteria (Ben Said et al., 2019; Sahoo et al., 2016). The dichotomy of target spectrum could well be related to r and k strategies. Narrow spectrum would be found in k‐selected microbiota, whereas broad spectrum would belong to r‐strategist. Therefore, to reconcile with ecological theory, bacteriocin applications in aquaculture should focus on narrow spectrum antimicrobials to help maintain a mature microbiota and avoid resistance development, while for food applications, broad‐spectrum bacteriocins would be more likely to succeed in the context of a pioneer microbiota where r‐strategist dominate. An additional advantage of bacteriocin is that so far, their mode of action and immunity appear to be unique to them (Ben Said et al., 2019). Nevertheless, bacteriocin resistance in foodborne pathogens has been reported and the development of combinatorial strategies that mitigate the risk of resistance emergence is recommended (Kumariya et al., 2019). Alternatively, fighting pathogens with strategies using heterospecific competitors that produce bacteriocins could effectively suppress resistance development (Bhattacharya, Stacy, & Bashey, 2019). Other, remaining challenges to address with bacteriocin applications in food are the undesirable impact on sensorial properties and the cost of production (Ben Said et al., 2019; Cifuentes Bachmann & Leroy, 2015).

Holistic microbial managing strategies of the rearing environment such as recirculating aquaculture systems (RAS) are meant for optimizing early fish microbiota ontogeny by stabilizing the water at low microbial carrying capacity and in other words by lowering resources and maximizing their use by highly specialized and competitive microbial strains for their resources. RAS relies on the ecological theory of r/K selection (MacArthur & Wilson, 1967). This theory relates to the assumption that, according to the stability of environmental conditions, either fast‐growing opportunist r‐strategists or K‐strategists will be selected. This theory was applied to microbial communities (Andrews & Harris, 1986), for which a stable environment with limited resources, therefore maximizing niche competition, will select for K‐strategists, whereas an unstable environment, rich in nutrients, low in niche competition, selects for r‐strategists; the second case being present in most aquaculture practices (Vadstein, Attramadal, Bakke, & Olsen, 2018). This approach is particularly well suited for the early developmental stages as egg microbiota ontogeny is highly influenced by environmental microbes (Bakke et al., 2019; Sylvain & Derome, 2017) and fish larvae are characterized both by immature immune and digestive systems at hatching. Eggs and fish larvae are thus particularly vulnerable to detrimental interactions with opportunistic (r‐strategists) microbes (Bakke et al., 2019). In addition, this approach can be theoretically applied to any fish host, as the microbial management is primarily modifying the rearing environment for long term (Vadstein et al., 2018), and not targeting one or few members of the microbial community in itself, as does most of the other microbial community managing tools (e.g. phages, probiotics, bacteriocins).

As an interesting application, in other livestock, probiotic use has been shown to improve meat quality (Vieco‐Saiz et al., 2019). Yet in fish, few studies have considered the effect of probiotic treatment from both the production and product quality perspective. Yang et al. (2019) evaluated the effects of *Bacillus cereus* as a probiotic on growth and fillet quality of Pengze crucian carp. They observed significant improvement in growth performance and flesh texture, driven by enhanced immunity and antioxidant capacity. However, the microbiological quality was not assessed, nor the shelf‐life, which would be an important criterion to consider. Indeed, the impact of probiotic treatment in aquaculture on the fish products is an important concern since the probiotic strains could turn into spoiling agents. *Lactobacillus*, *Lactococcus*, *Carnobacterium*, *Pseudomonas* and *Psychrobacter* strains have shown potential as probiotics in aquaculture (Vieco‐Saiz et al., 2019), but at the same time, bacteria belonging to these taxa have been reported as fish spoilage agents (Boziaris & Parlapani, 2017). An even more complex situation is the use of LAB, because they can be fish pathogens, as well as important spoilage agents, but they can also be bioprotective cultures. The fact that lactic acid bacteria can cause spoilage is a concern since these bacteria are often the focus of probiotic and bioprotective culture development. Their controversial role in spoilage has already been recognized, which stress the importance of considering strain‐specific traits (Leroi, 2010; Pothakos, Devlieghere, Villani, Björkroth, & Ercolini, 2015). For instance, LAB are often producers of biogenic amines in fermented foods. The metabolic pathway leading to the production of these toxic metabolites can support the primary metabolism in stressful environments and the strain‐specific occurrence pattern of decarboxylase genes suggest a role for horizontal gene transfer (Barbieri, Montanari, Gardini, & Tab anelli, 2019). Nevertheless, LAB have a food‐grade status, which makes them the most interesting candidates for food application development.

Being able to implement premortem strategies improving shelf‐life could be highly beneficial for the food industry. Can an aquaculture probiotic also be a bioprotective strain? There are three main strategies to isolate bioprotective strains for application in fish: use known safe strains with reported antimicrobial activity, isolate strains from spontaneously fermented food, or screening the autochthonous microbiota of fish (reviewed in Cifuentes Bachmann & Leroy, 2015) which is also a source of fish probiotics. Naturally occurring antimicrobial strains in fish ecosystems may be more competitive, but they are not necessarily suited for food preservation applications. For instance, some may produce biogenic amine, possess transferable antimicrobial resistance genes or display cytotoxic effects (Cifuentes Bachmann & Leroy, 2015). Moreover, if a probiotic strain producing a bacteriocin is used in aquaculture, resistance could develop overtime and this particular bacteriocin could lose its effect when used as a food additive on these products. Also, for probiotic applications in aquaculture, the choice of species and strain is critical since it is introduced at high doses. Indeed, *B. cereus* is a spore‐forming foodborne pathogen that can cause toxi‐infection when ingested at high doses. Although the probiotic strains used may not produce toxins, rapid genomic evolution such as horizontal gene transfer is always possible. Admittedly, probiotic properties, pathogenicity or spoilage activity can be strain‐specific attributes (Hossain, Sadekuzzaman, & Ha, 2017; Leroi, 2010; Remenant, Jaffrès, Dousset, Pilet, & Zagorec, 2015) which reduces the potential for conflicting applications. However, routine microbiological testing methods for quality control may not be specific enough to discriminate between a probiotic or a pathogen strain of the same species or genera.

Fish pathogens are different than foodborne pathogens, but since the aquaculture environment is a reservoir for human pathogens (Duman, Saticioglu, & Altun, 2019), integrated microbial modulation strategies targeting both human and fish pathogens could be developed. In support of this possibility, Kaktcham et al. (2017) isolated lactic acid bacteria from water and fish intestines and screened their antimicrobial activities against spoiling bacteria and pathogens (endogenous and exogenous). They demonstrate good potential, including the production of organic acids and some bacteriocins. The work of Anacarso et al. (2014) provides another example; a *Lactobacillus* strain was found active against both the fish pathogen *Aeromonas hydrophila* and the human pathogen *Listeria monocytogenes*. Thus, there is potential for carefully selected strains to act both as fish probiotics and food bioprotective culture.

How organisms are influenced by both biotic and abiotic factors, and how they contribute to ecosystem functions, is still an ongoing research question (Otwell et al., 2018). The evolutionary outcome of microbiota modulation strategies employed in aquaculture may have unforeseen repercussions in terms of resistance development but may also affect food quality and safety. Predicting such an outcome is further complicated by the fact that stochastic processes prevail in the primary succession stage (Dini‐Andreote, Stegen, van Elsas, & Salles, 2015), and microbiomes can exist in alternative stable states depending on the order of species arrival (Tipton et al., 2019). Some applications, such as probiotic or bioprotective strains use, will cause a perturbation of the host or food microbial community that reduces diversity (Vadstein et al., 2018). These may have the opposite of the intended effect if they leave the animal or product microbiota susceptible to pathogen growth. It is therefore essential that the consequences of the introduction of massive amounts of microorganisms or antimicrobials into the aquatic systems be evaluated from a system perspective, including from a food product quality and consumer health perspective. Yet, such studies that consider the impact on microbial communities from both aquaculture and food angles are still uncommon (Cao et al., 2019; Ceppa et al., 2018; Yang et al., 2019).

In general, strategies that result in lethal consequences, that is strong selective pressures, will more likely give rise to resistance. Gentle or highly targeted modulations of microbiomes may translate into more sustainable solutions over evolutionary timescales than drastic interventions such as broad‐spectrum antibiotic uses. However, because of the complexity of the ecological succession from the egg to the fork, the development of applications must also take into account the life history of the contaminating microbiota. For instance, the direct use of invariants such as bacteriocins may be well suited for food preservation applications but may not guarantee product safety if the same molecule was also present in the aquaculture environment, thereby selecting for resistance in the fish microbiota, including for future spoilage agents.

## 6 CONCLUSION

In the next five years, fish production is projected to increase by over 19% and fish farming is expected to exceed capture fisheries (OECD & FAO, 2015). Here, we showed how the selective context varies along the production chain. Understanding how microbiota respond and adapt to these changes in selective pressures is essential to fully develop the multiple applications reviewed herein that aim to modulate fish microbiota. Given that hundreds or fish species are commercially fished or farmed for food production around the world and consumed in various forms, a large fraction of our knowledge about microbial communities is context‐dependent. Performing system‐level studies to compare microbial relationships and dynamics patterns may help to grasp the relative importance of neutral versus non‐neutral forces in shaping microbial communities. For instance, microbial association network analyses are still scarce in both aquaculture and food microbiology studies. Carefully designed microbiota manipulation experiments in model systems may prove helpful to unravel the differences and similarities between fish hosts and products and how they respond to various applications. In fact, food microbiomes are easily manipulated and thus well‐suited models to investigate the assembly of microbial communities during the initial stage of ecological succession (Cocolin & Ercolini, 2015; Wolfe, Button, Santarelli, & Dutton, 2014). Similarly, the design of synthetic pioneering microbiota (mock communities) to colonize axenic/germ‐free fertilized eggs is a promising tool for developing sustainable management for inland/recirculated water systems in aquaculture. Finfish being particularly well‐suited organisms for gnotobiotic studies, germ‐free models were developed for various host species (reviewed in Lescak & Milligan‐Myhre, 2017).

Finally, to answer our initial question, “how can we harness evolutionary forces in the development of sustainable microbiota manipulation applications in the fish industry?” we conclude that integrated knowledge of the controlling abiotic and especially biotic factors is required. Combining the concepts and advances made in predictive microbiology with the knowledge of microbial ecology theory gained from model systems will help fill this gap to better understand how selective pressures can be balanced to develop and assess the risk of microbial manipulation strategies in the fish industry.

## CONFLICT OF INTEREST

None declared.

## Data Availability

Data sharing is not applicable to this article as no new data were created or analysed in this study.
